# Horizontally Transferred DNA in the Genome of the Fungus *Pyricularia oryzae* is Associated With Repressive Histone Modifications

**DOI:** 10.1093/molbev/msad186

**Published:** 2023-08-18

**Authors:** Natsuki Kobayashi, Thach An Dang, Kieu Thi Minh Pham, Luis B Gómez Luciano, Ba Van Vu, Kosuke Izumitsu, Motoki Shimizu, Ken-ichi Ikeda, Wen-Hsiung Li, Hitoshi Nakayashiki

**Affiliations:** Graduate School of Agricultural Science, Kobe University, Kobe, Japan; Graduate School of Agricultural Science, Kobe University, Kobe, Japan; Graduate School of Agricultural Science, Kobe University, Kobe, Japan; Department of Molecular and Cellular Biology, Baylor College of Medicine, Houston, TX, USA; Biodiversity Research Center, Academia Sinica, Taipei, Taiwan; Donald Danforth Plant Science Center, St. Louis, MO, USA; Graduate School of Agricultural Science, Kobe University, Kobe, Japan; Graduate School of Environmental Science, The University of Shiga Prefecture, Hikone, Japan; Department of Genomics and Breeding, Iwate Biotechnology Research Center, Kitakami, Japan; Graduate School of Agricultural Science, Kobe University, Kobe, Japan; Biodiversity Research Center, Academia Sinica, Taipei, Taiwan; Department of Ecology and Evolution, University of Chicago, Chicago, IL, USA; Graduate School of Agricultural Science, Kobe University, Kobe, Japan

**Keywords:** horizontal gene transfer, epigenetics, phytopathogenic fungi

## Abstract

Horizontal gene transfer (HGT) is a means of exchanging genetic material asexually. The process by which horizontally transferred genes are domesticated by the host genome is of great interest but is not well understood. In this study, we determined the telomere-to-telomere genome sequence of the wheat-infecting *Pyricularia oryzae* strain Br48. SNP analysis indicated that the Br48 strain is a hybrid of wheat- and *Brachiaria*-infecting strains by a sexual or parasexual cross. Comparative genomic analysis identified several megabase-scale “insertions” in the Br48 genome, some of which were possibly gained by HGT-related events from related species, such as *P. pennisetigena* or *P. grisea*. Notably, the mega-insertions often contained genes whose phylogeny is not congruent with the species phylogeny. Moreover, some of the genes have a close homolog even in distantly related organisms, such as basidiomycetes or prokaryotes, implying the involvement of multiple HGT events. Interestingly, the levels of the silent epigenetic marks H3K9me3 and H3K27me3 in a genomic region tended to be negatively correlated with the phylogenetic concordance of genes in the same region, suggesting that horizontally transferred DNA is preferentially targeted for epigenetic silencing. Indeed, the putative HGT-derived genes were activated when *MoKmt6*, the gene responsible for H3K27me3 modification, was deleted. Notably, these genes also tended to be up-regulated during infection, suggesting that they are now under host control and have contributed to establishing a fungal niche. In conclusion, this study suggests that epigenetic modifications have played an important role in the domestication of HGT-derived genes in the *P. oryzae* genome.

## Introduction

Pathogenic microbes are under strong natural selection pressure to counter the host immune system. Therefore, gaining new genetic variations is essential for the survival of pathogenic microbes. In addition to sexual reproduction, many phytopathogenic fungi seem to use the asexual exchange of genetic materials, such as parasexual recombination, horizontal gene transfer (HGT), and horizontal chromosome transfer (HCT), to produce genetic variation (Fitzpatrick 2012). Whole-genome sequencing projects in eukaryotes, especially in unicellular organisms, have revealed a significant contribution of HGT to genome evolution in eukaryotes (Keeling and Palmer 2008; Fitzpatrick 2012) as suggested previously in prokaryotes (Eisen 2000; Koonin, et al. 2001).

In fungi, various HGT-derived genes have been reported to serve as niche-determining factors since the 2000s (Temporini and VanEtten 2004; Friesen, et al. 2006; Milani, et al. 2012; Ambrose, et al. 2014). A well-known example is the gene encoding the virulence protein ToxA and its surrounding 14 kb sequence which was transferred from the wheat blotch pathogen *Stagonospora nodorum* to the wheat tan spot pathogen *Pyrenophora tritici-repentis* (Friesen, et al. 2006). This HGT event is believed to have occurred relatively recently, leading to the emergence of a new disease in wheat. More recently, extensive in silico analysis of fungal genome data identified possible HGT-derived genes, showing that HGT events among fungi or between fungi and distantly related organisms such as bacteria and Oomycetes have occurred more frequently than previously thought (Marcet-Houben and Gabaldon 2010; Richards, et al. 2011; Soanes and Richards 2014). Alexander et al. (2016) showed that up to 2% of genes in early diverged fungi, such as Microsporidia, were acquired by horizontal transfer from other organisms (Alexander, et al. 2016). Similarly, it has been reported that hundreds of genes may have been transferred from bacteria to Magnaporthales fungi (Zhang, et al. 2018). In addition, HGT may occur frequently between relatively closely related fungi. Qiu et al. (2016) reported that more than 90 genes could have been transferred between two Sordariomycete fungi, *Colletotrichum* and Magnaporthales. These findings indicate the important role of HGT in fungal genome evolution.

Genome integrity in eukaryotes may be maintained by epigenetic regulation systems, such as histone modifications and DNA methylation. Euchromatin is characterized by active gene expressions, relaxation of nucleosomes, high accessibility to transcription machinery, and specific histone modifications, such as acetylation of histones and di- and tri-methylation of H3 lysine 4 (H3K4me2/3) (Li, et al. 2007). In contrast, gene expression is repressed in constitutive heterochromatin, which is characterized by condensed nucleosomes, dense cytosine methylation, and histone marks, such as H3K9me3 (Li, et al. 2007). In addition, there is an intermediate state, called facultative heterochromatin, in which genes are reversibly repressed depending on environmental or developmental conditions. H3K27me3 is a hallmark of facultative heterochromatin in many fungal species (Erlendson, et al. 2017). These epigenetic compartments can affect various aspects of genome regulation, such as gene expression, DNA repair, and mutation rates (Li, et al. 2007; Habig, et al. 2021). How HGT-derived genes are integrated into the epigenetic genome regulation system is an interesting question, but knowledge of this process is very limited.

*Pyricularia oryzae* (syn. *Magnaporthe oryzae*) is a phytopathogenic fungus that causes blast disease in various gramineous plants such as rice, wheat, oat, and finger millet (Kato, et al. 2000). *P. oryzae* strains that are parasitic on different host plant species are genetically distinguishable and are called pathotypes. Examples include the *Oryza* pathotype (MoO), *Eleusine* pathotype (MoE), *Lolium* pathotype (MoL), and *Triticum* pathotype (MoT). Among them, wheat blast (MoT) has recently become a global threat, as it spreads from South America to wheat-producing countries in Eurasia (Islam, et al. 2016) and Africa (Tembo, et al. 2020). Since the genome sequencing of the 70-15 strain (laboratory strain largely derived from MoO Guy11) in 2005 (Dean, et al. 2005), the genomes of various *P. oryzae* strains and related species have been completely or partially sequenced (Yoshida, et al. 2016; Gómez Luciano, et al. 2019 ; Peng, et al. 2019; Rahnama, et al. 2020), serving as model plant pathogens for comparative genomics.

In this study, we first determined the telomere-to-telomere genome sequence of Br48, which is an MoT strain isolated in Brazil in 1990. We then used a comparative genomics approach and developed an index, the “Index of Phylogenetic Concordance (IPC)”, to identify HGT candidates in the Br48 genome. Finally, we conducted ChIP-seq and RNA-seq analysis to see whether HGT candidates tend to be located in genomic regions marked with silent histone modifications such as H3K9me3 and H3K27me3, and how these candidates are epigenetically regulated. Our aim was to increase our understanding of how horizontally transferred genes become adapted or domesticated in the host genome.

## Results

### Nearly Telomere-to-Telomere Genome Assembly of the Wheat Infecting Strain, Br48

Br48 is a wheat-infecting strain of *P. oryzae* collected in Brazil in 1990 (Urashima, et al. 1993). This strain belongs to the ancient MoT population that emerged in South America in the 1980s (Igarashi, et al. 1986), and therefore, serves as a model to gain insight into the origin of MoT. In this study, whole-genome shotgun sequencing was used to generate a near-complete genome assembly of the Br48 strain using PacBio, HiSeq, and MiSeq platforms (supplementary table S1, Supplementary Material online). The initial draft assembly was built from 3.5 GB of PacBio long reads using the Hierarchical Genome Assembly Process (HGAP) at DDBJ. Assembly polishing was performed using the Illumina reads. The resulting unitigs were joined using contigs constructed from the HiSeq and MiSeq reads. In addition, long polymerase chain reaction (PCR)-based verification was performed to fill gaps with reference to *P. oryzae* genomes in public databases. The final size of the assembled Br48 genome was approximately 42.5 Mb in seven chromosomes with 12,745 protein-coding genes, which is comparable to previously sequenced *P. oryzae* isolates. Each chromosome was named based on the MG8 genome assembly of the 70-15 strain. Telomere repeat sequences and/or *M. oryzae* telomeric retrotransposons (MoTeRs) (Starnes, et al. 2012) were found at both ends of each chromosome, with the exception of one end of chromosome 1, where the rRNA cluster exists, indicating that the Br48 genome assembly is nearly telomere-to-telomere. In public databases, a nearly complete genome sequence is available for four *P. oryzae* strains, 70-15 (*Oryza* pathotype, MoO), MZ5-1-6 (*Eleusine* pathotype, MoE) (Gómez Luciano, et al. 2019), LpKY97 (*Lolium* pathotype, MoL) (Rahnama, et al. 2020), and B71 (*Triticum* pathotype, MoT) (Peng, et al. 2019). Our Br48 assembly showed global colinearity for each chromosome with the *P. oryzae* genome assemblies, especially those of the B71 and LpKY97 strains (supplementary fig. S1, Supplementary Material online).

### Contribution of a *Brachiaria* Strain to the Emergence of Br48 as a Wheat Pathogen

**Fig. 1.**
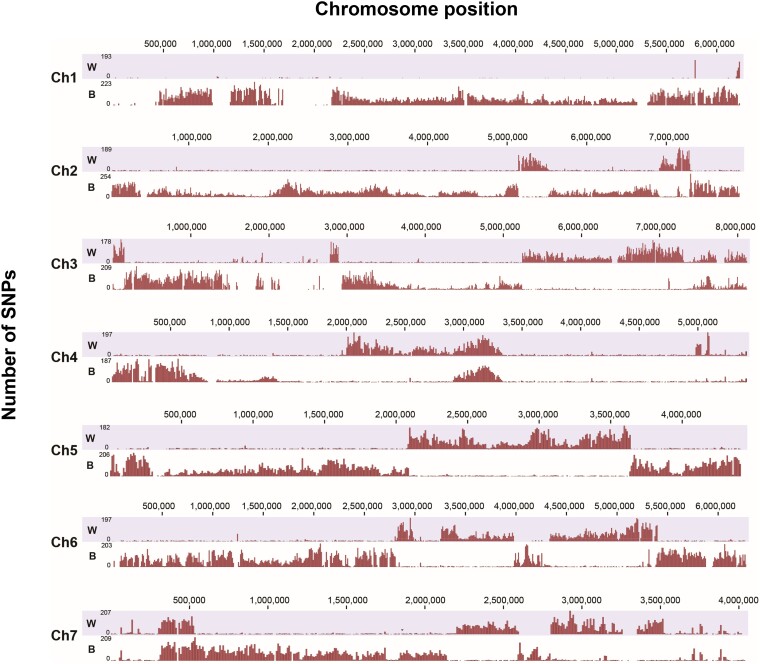
SNP distribution on the seven Br48 chromosomes. For each chromosome, the lower and upper panels show SNPs identified by comparison of the Brachiaria strain Br35 (*B*) and a hypothetical wheat strain (*W*) consisting of chromosomes 2 and 5 from Br116.5 (wheat strain) and the other chromosomes from Br118.2 (another wheat strain), respectively.

Inoue et al. (2017) suggested that the direct ancestor of Br48 inherited a 1.6-Mb chromosomal segment on chromosome 5 from a *Brachiaria* strain. This event is crucial for Br48 to be a wheat-infecting strain because the 1.6-Mb segment carries the virulent B-type *PWT3* allele, which may have replaced the avirulent A-type *PWT3* allele in an ancestor of wheat blast fungi. To examine how the 1.6-Mb segment was introduced into Br48, whole-genome SNP analysis was performed in a *Brachiaria* strain (Br35). The SNP distribution on chromosome 5 suggested that chromosome 5 of Br48 comprises two distinct parts; the major part (includes a 2.1-Mb and a 0.7-Mb segment) that are composed of sequences very closely related to a Br116.5 (a wheat isolate) and LpKY97 (supplementary fig. S2, Supplementary Material online), respectively, while the minor part is a 1.6-Mb segment that shows higher sequence similarity with Br35, indicating that the Br48 genome is generated by chromosomal recombination between an MoT or MoL strain and a *Brachiaria* strain (fig. 1). In support of this hypothesis, all Br48 chromosomes were generally segmented with regions having a very high sequence similarity with either a wheat strain or a *Brachiaria* strain, which is likely the result of a chromosomal crossover (fig. 1). These findings indicate that Br48 is a hybrid between an MoT or MoL strain and a *Brachiaria* strain. Thus, *P. oryzae* may undergo genetic recombination under natural conditions by a sexual cross or parasexual recombination through anastomosis (Noguchi, et al. 2006), which may lead to the birth of a new pathotype.

### Megabase-Scale Variations in *Pyricularia oryzae* Genomes due to HGTs From Related *Pyricularia* Species

**Fig. 2.**
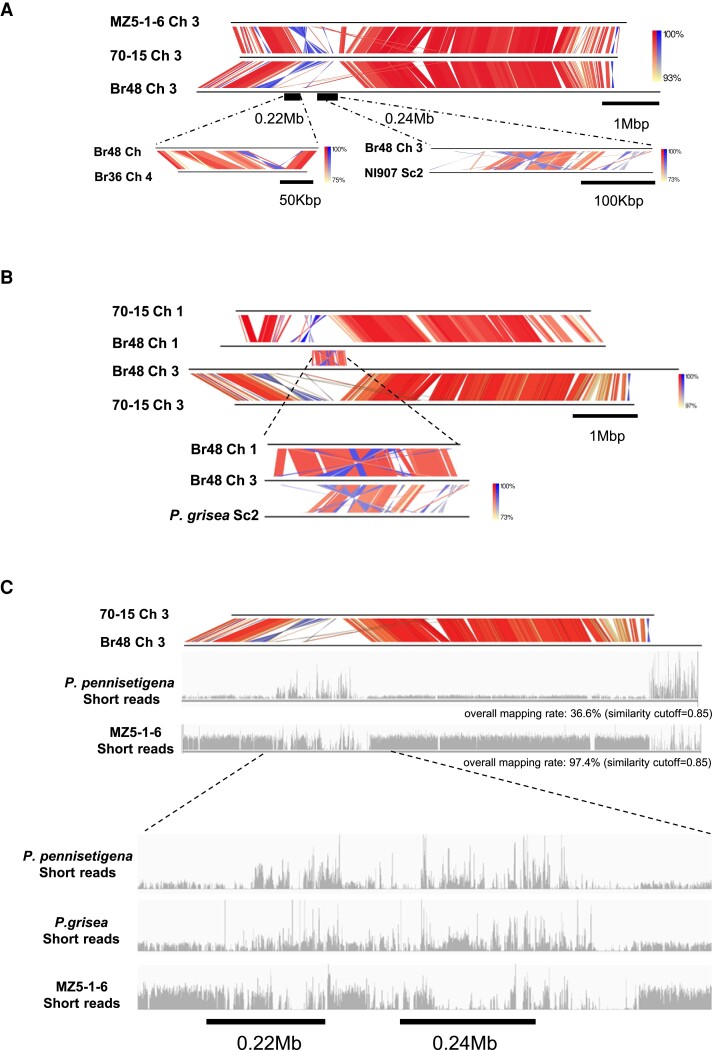
Megabase-scale variations in *P. oryzae* genomes possibly involved HGT events from related *Pyricularia* species. (*A*) Genome segments of *P. grisea* (Dig41, scaffold 2_#247,800-#564,400) and *P. pennisetigena* (Br36, Ch4_#580,000-#732,400) that were syntenic to large genome segments of Br48 (Ch3_#1,526,800-#1,727,500 and #2,120,000-#2,427,000) that have no corresponding region in the 70-15 genome. (*B*) The genome segment syntenic to a *P. grisea* genome segment was duplicated on chromosomes 1 (#1,685,300-#1,965,600) and 3 (#2,120,000-#2,427,000) with rearrangements in Br48. (*C*) Short reads of *P. pennisetigena*, *P. grisea*, and *P. oryzae* (MZ5-1-6) were mapped to chromosome 3 of Br48 with a similarity cutoff of 0.85. Read mapping in the megabase-scale rearranged region is shown in a close-up view. Color gradient in genome comparison representing the identities of the BLAST hits. Red for matches in the same direction and blue for inverted matches.

To gain insight into the evolutionary history of the *P. oryzae* genome, we focused on megabase-scale variations among the five *P. oryzae* genomes. Peng et al. (2019) reported two large genomic rearrangements in the B71 genome relative to the reference strain 70-15 (Peng, et al. 2019). First, part of chromosome 1 of 70-15 was translocated to the end of B71 chromosome 6 in the opposite orientation, and second, a 1.3-to-2.9 Mb sequence on chromosome 3 of B71 was lost in 70-15. The former rearrangement was observed in all *P. oryzae* strains examined except 70-15, so it is likely attributable to a 70-15-specific rearrangement. The latter rearrangement was also commonly observed in the Br48, B71, and LpKY97 genomes, but the MZ5-1-6 genome showed a different rearrangement pattern (fig. 2*A*). A detailed sequence comparison of the rearrangement in chromosome 3 between Br48 and 70-15 revealed that a 169,959 bp segment (#1,567,826–# 1,737,784 nt) in 70-15 was expanded to 1,427,810 bp in Br48, indicating that a total of ∼1.25 Mb sequences were gained in Br48 or lost in 70-15. A search for homologous sequences in various *P. oryzae* isolates in the public databases found two segments (∼0.22 Mb and ∼0.24 Mb) in the expanded region of Br48 that were syntenic with those in chromosome 4 (Scaffold 6) of *P. pennisetigena* (Br36) and Scaffold_2 of *P. grisea* (NI907), respectively (fig. 2*A*), whereas such syntenic regions were not detectable in any chromosome of 70-15. In MZ5-1-6, not the whole but only parts of the 0.22 Mb and 0.24 Mb segments were detected on chromosomes 3 and 7. Interestingly, the sequences shared high similarity with the 0.24 Mb segment were also identifiable on chromosome 1 of Br48, while the corresponding region was missing in both 70-15 and MZ5-1-6 (fig. 2*B*, supplementary fig. S1, Supplementary Material online). These data imply that the rearrangements of chromosomes 1 and 3 of Br48 were associated with duplication or two insertional events.

In addition to the genomic rearrangements mentioned above, the MoT strains (Br48 and B71) possess an approximately 0.8 Mb “extension” at one end of chromosome 3, with reference to the other *P. oryzae* strains used in this study (fig. 2*A*, supplementary fig. S1, Supplementary Material online). The sequence in the extension shared partial similarities with the mini-chromosome found in LpKY97. Thus, as suggested previously (Peng et al. 2019), the “extension” could be the result of a mini-chromosome addition to the end of chromosome 3 with complex rearrangements. Specific to the Br48 genome, “inserted” segments of 0.36 and 0.21 Mb were detectable on chromosomes 6 and 7, respectively (supplementary fig. S1, Supplementary Material online). The 0.36 Mb segment on chromosome 6 showed partial sequence similarity with the rearranged region on chromosome 3 and a mini-chromosome found in the B71 strain. The 0.21 Mb segment on chromosome 7 also showed partial similarity with the rearranged region on chromosome 3. Interestingly, the three possibly inserted sequences, the 0.36 Mb segment on chromosome 6, the 0.21 Mb segment on chromosome 7, and the 0.22 Mb segment on chromosome 3, shared a stretch of 40 kb homologous sequences that showed synteny with a segment on chromosome 4 of *P. pennisetigena* (supplementary fig. S3, Supplementary Material online). These results indicate that the “inserted” segments in the Br48 genome may share common origins, or parts of which have undergone multiple translocations and/or duplications.

To gain further insight into the rearranged regions, short reads of whole-genome shotgun sequences of *P. pennisetigena*, *P. grisea*, and *P. oryzae* (MZ5-1-6) were mapped to the Br48 genome. Before mapping, reads derived from transposable elements (TEs) identified in the Br48 genome (supplementary table S2, Supplementary Material online) and rRNA clusters were removed to exclude bias from the difference in abundance between isolates. With a similarity cutoff of 0.85, 36.6%, 35.1%, and 97.4% of reads from *P. pennisetigena*, *P. grisea,* and MZ5-1-6, respectively, were mapped to the Br48 chromosomes. As shown in figure 2*C*, the abundance of mapped MZ5-1-6 reads on chromosome 3 was generally high but considerably lower in the rearranged regions, while that of *P. pennisetigena* and *P. grisea* was considerably higher in those regions despite their overall low mapped rates, suggesting that the sequences in the rearranged regions had a higher similarity to those in *P. pennisetigena* and *P. grisea*.

Similarly, overall nucleotide substitution rates of MZ5-1-6 and Dig-41 (*P. grisea*) against Br48 were 0.7% and 6.7%, respectively, while those in the 0.22 Mb and 0.24 Mb regions on chromosome 3 were 7.3% and 12.1% in MZ5-1-6, respectively, and 6.9% and 9.1% in Dig-41, respectively. Thus, the sequences in the 0.22 Mb and 0.24 Mb regions in Br48 have higher similarities to those in *P. grisea* than to those in *P. oryzae* (MZ5-1-6), contrary to the overall higher sequence similarity between MZ5-1-6 and Br48. It should be noted that in these regions, frequent repeat-induced point mutation (RIP)-like mutations that elevated the substitution rates were detected likely due to the presence of repeated/duplicated sequences (Ikeda, et al. 2002; Peng, et al. 2019). The ratio of RIP-like substitutions (C/G to T/A) in total SNPs between Br48 and Dig-41 increased from 26.6% in the whole genome to 41.8% in the 0.22Mb region and to 51.4% in the 0.24Mb region. These data are consistent with the idea that the “inserted” and “extended” segments were horizontally transferred, at least partly, from related *Pyricularia* species such as *P. pennisetigena* or *P. grisea* at certain time points after the diversification of *P. oryzae* from these species.

### ‘Mobile Genes’ are Often Phylogenetically Discordant

As described above, the 0.22 Mb and 0.24 Mb segments on chromosome 3 were possibly horizontally transferred from other *Pyricularia* species, and parts of these sequences were possibly translocated to the “inserted segments” on other chromosomes. A detailed sequence analysis of these segments revealed that the 0.24 Mb segment contains a large imperfect inverted repeat (IR) sequence of 0.1 Mb long. In the first half of the 0.1 Mb segment, 18 protein-coding genes (mostly pseudogenes) were identifiable. These sequences were repetitive because they have homologs in the latter half and also additional homologs on chromosome 1. Consequently, these genes suffer from frequent RIP-like mutations as mentioned above. In addition, approximately 30 genes are recognizable outside the IR sequence in the 0.24 Mb segment. Notably, most of the genes in the 0.24 Mb segment showed the highest similarity to genes in *Pyricularia* species other than *P. oryzae* (supplementary table S3, Supplementary Material online).

**Fig. 3.**
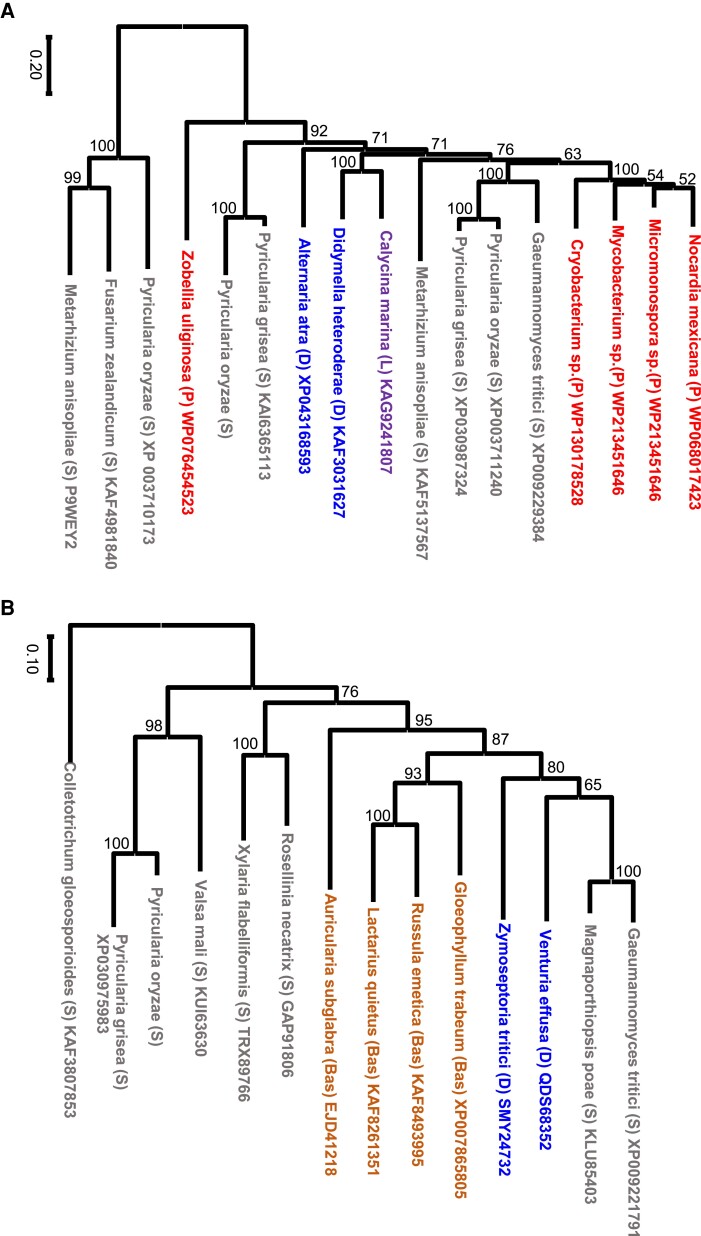

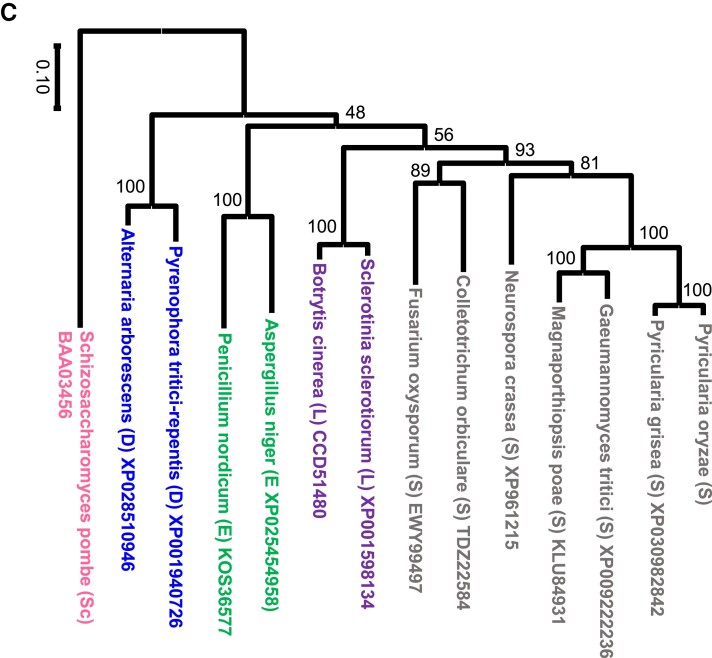
Neighbor-joining trees of two genes in the “inserted” segments in the Br48 genome, and that of a conserved gene. (*A*) FAD/FMN-binding dehydrogenase. (*B*) Putative small secreted protein. (*C*) Elongation factor-1 gamma (the conserved gene). Accession numbers of protein sequences used in the analysis are shown after species names except sequences detected in the segment of Br48. Accession numbers of protein sequences used in the analysis are shown after species names. Bas, Basidiomycetes (shown in Brown); E, Eurotiomycetes (green); D, Dothideomycetes (Blue); L, Leothiomycetes (purple); P, Prokaryotes (red); S, Sordariomycetes (gray); Sc, Schizosaccharomyces (pink).

Phylogenetic analysis of these genes revealed that, in many cases, their gene trees were incongruent with the species tree, and some of them were grouped with genes from distantly related organisms, such as bacteria and basidiomycetes. The phylogenetic trees of two striking examples, FAD/FMN-binding dehydrogenase, and a putative small secreted protein are shown together with those of the well-conserved elongation factor-1 gamma sequences in figure 3*A*–*C* (supplementary fig. S4, Supplementary Material online). In figure 3*C*, Sordariomycetes (S), to which *Pyricularia* belong, are most closely related to Leothiomycetes (L), followed by Eurotiomycetes (E) and Dothideomycetes (D), and most distantly related to Schizosaccharomycetes (Sc) within ascomycete fungi. However, in the phylogenetic tree of FAD/FMN-binding dehydrogenase-like genes (fig. 3*A*), sequences from various ascomycete fungi did not follow the species tree, and genes from bacteria such as *Nocardia* and *Micromonospora* clustered with *Pyricularia* and *Gaeumannomyce* genes.

In the second example, genes encoding a putative small secreted protein in ascomycetes also did not form a phylogenetic tree consistent with the species phylogeny (fig. 3*B*). Moreover, genes from basidiomycetes were grouped within the ascomycete genes. These data suggest that the “mobile segments” contained genes that had been horizontally transferred.

**Fig. 4.**
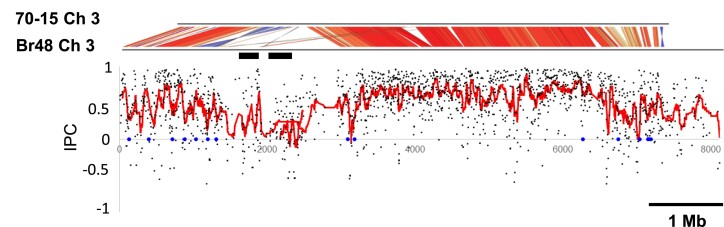
IPC decreases in genomic segments showing structural variations between the 70-15 and Br48 strains. Black dots represent the IPC values of individual genes. Red dots indicate moving averages of 15 consecutive IPC values. Black bars show the positions of the 0.22 Mb (left) and 0.24 Mb (right) regions, respectively. The genomic positions of *P. oryzae* homologs to possible HGT-derived genes identified by Qiu et al. (2016) are shown as blue dots.

To globally detect possible horizontally transferred genes in the Br48 genome, we examined the phylogenetic concordance of genes by defining an index, namely the IPC. The index was calculated as follows: twenty ascomycete species were selected and ranked according to their species similarity to *P. oryzae* based on protein sequences of multiple conserved genes. Next, possible orthologs of a *P. oryzae* gene were identified in these fungal species using a BLASTP search and ranked according to their BLAST scores (max scores). The IPC of a gene is given as a correlation coefficient between the two rankings based on species similarity and the BLAST score. As shown in figure 4, the values of IPC were drastically reduced in the 0.22 Mbp and 0.24 Mbp segments that likely contained HGT-derived genes, suggesting that IPC can function as an indicator of HGT-derived genes. Interestingly, IPC values tended to decrease in genomic regions where sequences are not well-conserved between Br48 and MoO (70-15) including the “inserted” and “expanded” segments on chromosome 3 (fig. 4). This tendency was also observed for other chromosomes (supplementary fig. S5, Supplementary Material online).

To test IPC for identifying the locations of HGT candidates, we used a list of genes that are supposed to be horizontally transferred from *Colletotrichum* to *Magnaporthiopsis incrustans* (Qiu et al. 2016). We identified 92 possible orthologs of the 93 *M. incrustans* genes in Br48 (supplementary table S4, Supplementary Material online). The genomic positions of these genes were associated with a decrease in IPC on chromosome 3 (fig. 4) as well as on chromosomes 6 and 7 (supplementary fig. S5, Supplementary Material online), which supports the validity of IPC.

### Tight Association of Silent Epigenetic Marks With Variable Domains in the Br48 Genome

**Fig. 5.**
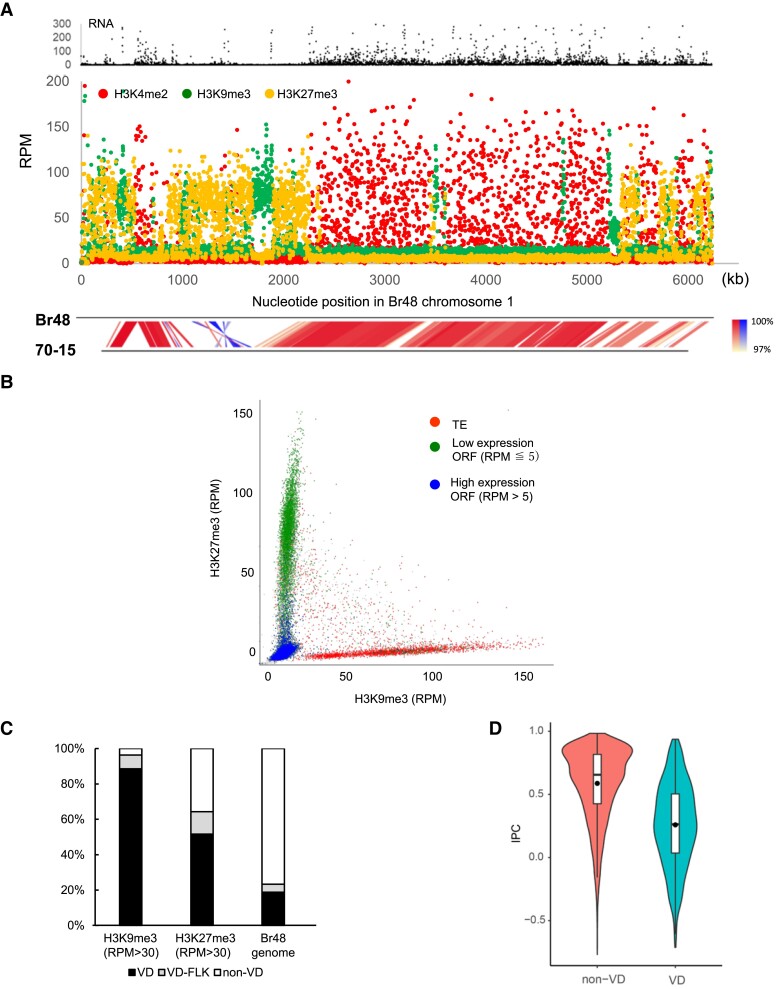
Silent epigenetic marks are tightly associated with variable domains (VDs) in the Br48 genome. (*A*) Read mapping data of RNA-seq and ChIP-seq analysis on chromosome 1 of Br48. RNA used in the analysis was extracted from vegetative mycelia. Reads mapped to TE sequences (supplementary table S2, Supplementary Material online) were collected and then mapped to the Br48 genome. Each dot indicates an RPM value of a 1 kb segment in the Br48 genome (see the text). (*B*) Relationship between H3K9me3 and H3K27me3 levels at 1 kb resolution. RPM values of H3K9me3 (X-axis) and H3K27me3 (Y-axis) in a 1 kb segment were plotted as gray dots. Red dots indicate TE-containing 1 kb segments (more than 50% of the sequence is covered by TE reads). Green dots show silent open reading frame (ORF)-containing 1 kb segments (RNA RPM is less than 5, and more than 50% of the sequence corresponds to exons). Blue dots indicate active ORF-containing 1 kb segments (RNA RPM is larger than 5, and more than 50% of the sequence corresponds to exons). (*C*) Proportions of H3K9me3-rich (RPM >30) and H3K27me3-rich (RPM >30) segments in the three genome domains, VD, 3 kb sequences adjacent to VDs (VD-FLK), and non-VDs (see details in the text). (*D*) Violin plots of IPC values in VDs and non-VDs. The horizontal line and closed circle in the boxplot represent the median and the average, respectively.

To obtain a view of the epigenetic landscape of the Br48 genome, we performed RNA-seq and chromatin immunoprecipitation (ChIP)-seq analyses of vegetative mycelia using antibodies against three major histone modifications, H3K4me2, H3K9me3, and H3K27me3. For each analysis, the reads per million mapped reads (RPM) was calculated using a 1 kb window throughout the chromosomes. Figure 5*A* shows the ChIP-seq and RNA-seq data for chromosome 1. In general, the level of RNA expression was positively associated with that of H3K4me2 and negatively associated with those of H3K9me3 and H3K27me3, as reported for various organisms (Li, et al. 2007) (fig. 5*A*, supplementary fig. S6, Supplementary Material online). However, while their association was evident in a global view, the correlation between RNA and H3K4me2 levels was not significant (R^2^ = 0.0055) when examined at 1 kb resolution (supplementary fig. S7, Supplementary Material online). Thus, H3K4me2 may not activate gene expression directly but rather act as a marker of chromosomal domains where genes are primed to be expressed.

In contrast, H3K9me3 and H3K27me3 were tightly associated with low gene expression even at a 1 kb resolution (supplementary fig. S7, Supplementary Material online). H3K9me3 and H3K27me3 also formed global-enriched domains on chromosomes but to a lesser extent than H3K4me2. H3K4me2- and H3K9me3-rich domains were almost exclusive of each other, which may correspond to euchromatin and constitutive heterochromatin, respectively. H3K9me3-rich and H3K27me3-rich domains often occurred at similar chromosomal locations, such as telomeres and subtelomeres. However, at 1 kb resolution H3K9me3 and H3K27me3 mostly did not overlap with each other (fig. 5*C*). Only 3.08% of the H3K27me3-rich segments (>30 RPM) showed high H3K9me3 levels (>30 RPM), suggesting that these silent histone marks were differently regulated.

As TEs are known to induce heterochromatin formation at and around the site of their integration, the association of TEs with silent histone marks was examined. First, we created a non-redundant list of repeat sequences comprising 21 TEs including DNA transposons and retrotransposons, 13 short repeats, and 8 unknown repeats (supplementary table S2, Supplementary Material online). We defined TEs by their characteristic genes, such as reverse transcriptase and transposase, and short repeats by a unit of repeated sequences within a range of 100–300 bp. As shown in figure 5*B*, TEs were mostly found in H3K9me3-rich domains, even though a minority of them were marked with H3K27me3 or both H3K9me3 and H3K27me3. The H3K27me3-rich domains were associated with lowly expressed genes (RPM ≤ 5), while highly or moderately expressed genes (RPM > 5) largely resided in genomic regions without H3K9me3 and H3K27me3 or sometimes in regions only with H3K27me3 (fig. 5*B*).

Notably, the silent histone marks seemed to be tightly associated with genomic locations showing a structural variation between the Br48 and 70-15 strains such as “inserted” and “expanded” segments in the Br48 genome (fig. 5*A*; supplementary fig. S8, Supplementary Material online). To address this point, we collectively called them “variable domains (VDs),” defined as genome segments over 2 kb in size that does not have a homologous counterpart in the 70-15 genome when two chromosomes are aligned using the BLAST algorithm. VDs likely result from hypermutation, insertion, interchromosomal translocation, and transposition in Br48, or hypermutation and deletion in 70-15. The variable domain (VD) sequences accounted for 18.8% of the Br48 genome (table 1). Furthermore, 88.6% and 51.6% of the 1 kb segments showing high H3K9me3 and H3K27me3 levels (RPM > 30), respectively, were found in VDs (fig. 5*C*). When the 3 kb sequences adjacent to VDs were included in the analysis [flanking sequences to VD (VD-FLK)], the percentages of the H3K9me3-rich and H3K27me3-rich segments increased to 96.4% and 64.3%, respectively (fig. 5*C*). Thus, almost all high H3K9me3 segments and approximately two-thirds of high H3K27me3 segments were associated with VDs. Consequently, 37.8%, 45.9%, and 2.9% (86.6% in total) of the 1 kb segments in VDs showed high levels (RPM > 30) of H3K9me3, H3K27me3, and both, respectively.

**Table 1.** H3K9me3- and H3K27me3-Enriched Segments in the Br48 Genome.

	VD	VD-FLK	Non-VD	Total
**Total genome**				
segments	8,006	1,896	32,600	42,502
%	18.8%	4.5%	76.7%	
**H3K9me3-rich (RPM >30)**				
segments	3,254	286	134	3,674
%	88.6%	7.8%	3.6%	
**H3K27me3-rich (RPM >30)**				
segments	3,904	959	2,703	7,566
%	51.6%	12.7%	35.7%	

VD, variable domain (see text); VD-FLK, 3 kb flanking sequences to VD.

We also examined the relationship between VDs and a decrease in IPC as mentioned above. In the VD and non-VD domains, 618 and 8,266 genes, respectively, had an IPC value. The averages of IPC values in VDs and non-VD domains were 0.258 and 0.587, respectively, which differed significantly (*P* = 1.8e-155, *t*-test, fig. 5*D*).

### Association of H3K9me3 and H3K27me3 Marks With Sequences of “Movement”

To study the factors that induce silent histone modifications in VDs, the involvement of TEs was first examined. In the Br48 genome, 8.3% of the 1 kb segments contained TE sequences, whereas in VDs, the proportion increased to 33.1%. We performed enrichment analysis using three criteria: TE, TE-flanking (3 kb), and non-TE segments (table 2). In both VD and non-VD regions, TE-segments were highly enriched in H3K9me3-rich segments compared to the average, suggesting that TEs induced H3K9me3. In contrast, in H3K27me3-rich segments, TE segments were only slightly enriched in VDs or depleted in non-VDs, suggesting that the enrichment of H3K27me3 in VDs was not directly related to the abundance of TEs in the segments. Notably, H3K27me3-rich segments were detected more frequently in TE-flanking segments than in TE-segments, regardless of VDs or non-VD regions (table 2).

**Table 2.** Association of TE With Silent Histone Marks in the Br48 Genome.

	VD			Non-VD			Total		
	TE	TE-FLK	Non-TE	TE	TE-FLK	Non-TE	TE	TE-FLK	Non-TE
**Total genome**									
Segments	2,649	2,046	3,311	875	2,712	30,909	3,524	4,758	34,220
%	33.1%	25.6%	41.4%	2.5%	7.9%	89.6%	8.3%	11.2%	80.5%
Enrichment	3.99	2.28	0.51	0.31	0.70	1.11			
**H3K9me3-rich (RPM >30)**									
Segments	1,987	660	607	390	9	21	2,377	669	628
%	61.1%	20.3%	18.7%	92.9%	2.1%	5.0%	64.7%	18.2%	17.1%
Enrichment	7.36	1.81	0.23	11.20	0.19	0.06	7.80	1.63	0.21
**H3K27me3-rich (RPM >30)**									
Segments	570	1,191	2,143	84	449	3,129	654	1,640	5,272
%	14.6%	30.5%	54.9%	2.3%	12.3%	85.4%	8.6%	21.7%	69.7%
Enrichment	1.76	2.73	0.68	0.28	1.10	1.06	1.06	1.94	0.87

VD, variable domain (see text); TE-FLK, 3 kb flanking sequences to TE.

**Fig. 6.**
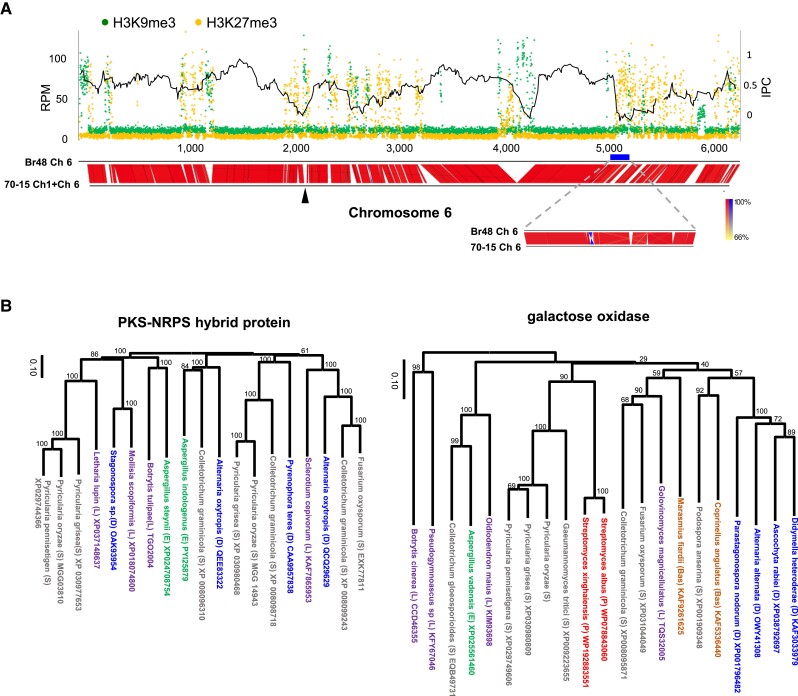
Association of silent histone marks with sequences of “movement”. (*A*) Read mapping of H3K9me3 and H3K27me3 on chromosome 6 of Br48. The black line shows moving averages of 15 consecutive IPC values on chromosome 6. The thick blue line indicates the H3K27me3-rich 110 kb region analyzed in detail (see the text). Since a part of chromosome 6 in the 70-15 strain was translocated to chromosome 1, the corresponding region in chromosome 1 was added to chromosome 6 of the strain for a comparison of genome structure. The black arrowhead indicates the connection point. (*B*) Neighbor-joining trees of PKS-NRPS hybrid protein and galactose oxidase protein in the H3K27me3-rich 110 kb region. Accession numbers of protein sequences used in the analysis are shown after species names. Bas, Basidiomycetes (shown in brown); E, Eurotiomycetes (green); D, Dothideomycetes (blue); L, Leothiomycetes (purple); P, Prokaryotes (red); S, Sordariomycetes (gray).

These data are consistent with figure 5*B*, suggesting that H3K27me3 is associated more with low-expression genes than with TEs. Therefore, we next focused on the relationship between H3K27me3 and HGT candidates. As shown in figure 6*A*, H3K27me3-rich regions in the genome were associated with decreased IPC values on chromosome 6. This trend was also observed on other chromosomes (supplementary fig. S9, Supplementary Material online), supporting the idea that H3K27me3 is associated with HGT-derived genes.

Notably, some stretches of genomic regions, such as those on chromosomes 2, 5, and 6, were found to be H3K27me3-rich but did not correspond to either a subtelomeric region or any location of large rearrangements, and were even free of TEs (supplementary fig. S6, Supplementary Material online). Thus, we focused on one such region on chromosome 6 (fig. 6*A*). This region was approximately 110 kb in length (nucleotide #5,091,400 to #5,200,100) and was mostly well aligned to the corresponding genomic region in the 70-15 strain with a few small gaps (fig. 6*A*).

The above region of Br48 contains 24 predicted protein-coding genes that show several notable features (supplementary table S5, Supplementary Material online). First, as indicated by a decrease in IPC, phylogenetic relationships of these genes are often discordant with fungal species phylogeny but were almost always concordant within Magnaporthales. This contrasts with the genes in the large rearrangement regions on chromosomes 1 and 3, in which many of the resident genes were sometimes phylogenetically discordant, even within Magnaporthales. For example, the phylogenetic trees of the polyketide synthase (PKS)-nonribosomal peptide synthetase (NRPS) hybrid protein and the galactose oxidase/kelch repeat protein are shown in figure 6*B*. This region contains two genes encoding a PKS–NRPS hybrid protein (orthologs of MGG_03810 and MGG_14943). In most cases, PKS-NRPS homologs from different classes of fungal species are clustered together. Notably, no ortholog of MGG_03810 was found in the representative plant pathogenic Sordariomycete fungi, *Colletotrichum* and *Fusarium*, whereas its orthologs were found in other classes of fungi such as Leotiomycetes. Regarding galactose oxidase/kelch repeat proteins (orthologs of MGG_03826), homologs of MGG_03826 were detected in a wide range of organisms, including Ascomycetes, Basidiomycetes, and Actinomycetes. The amino acid sequence similarity and identity between the homologs in Br48 and *Streptomyces albus* were 62.6% and 50.5%, respectively. This notable sequence similarity between distantly related organisms and sequence conservation within Magnaporthales suggests the possible involvement of HGT before the diversification of Magnaporthales fungi.

Second, this region exhibited the typical characteristics of a secondary metabolite biosynthetic gene cluster (smBGC). At least 16 of the 24 genes were thought to be involved in secondary metabolite synthesis, exemplified by the PKS-NRPS hybrid protein, P450, terpene synthase, etc. (supplementary table S5, Supplementary Material online). It is often suggested that smBGC is acquired via HGT (Khaldi, et al. 2008; Wisecaver and Rokas 2015). This H3K27me3-enriched region may also be the case.

### Transcriptional Regulation of HGT-Candidates by H3K27me3

**Fig. 7.**
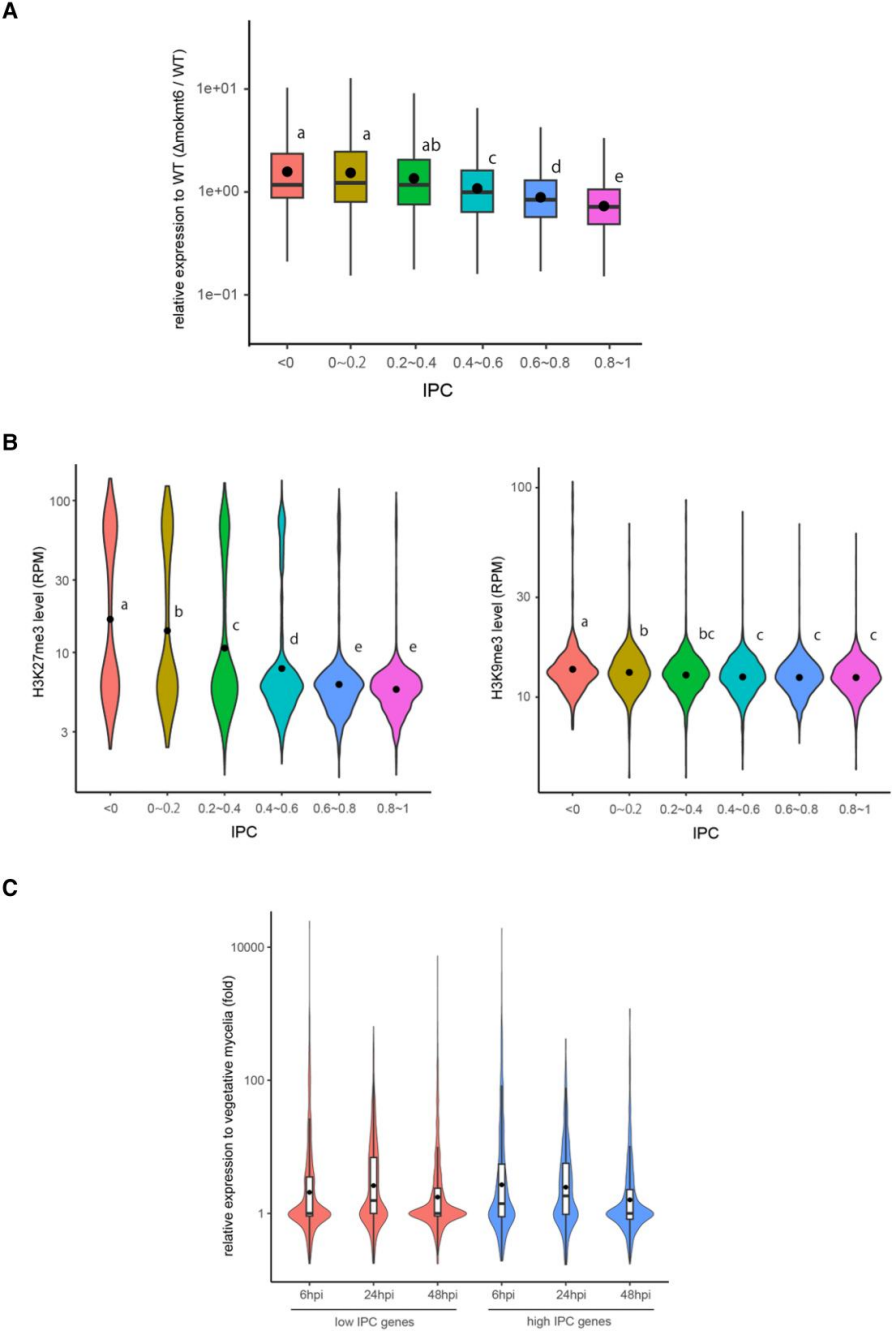
Transcriptional regulation of HGT-candidates by H3K27me3. (*A*) The boxplots show the transcriptional changes in vegetative mycelia of Δmokmt6 relative to wild-type in six gene groups categorized by IPC levels. Different letters indicate statistically significant differences (Tukey’s HSD test on logarithmically transformed data, *P* ≤ 0.01). (*B*) The violin plots show the levels of H3K27me3 (left) and H3K9me3 (right) in six gene groups categorized by IPC levels. The closed circle represents the average. Different letters indicate statistically significant differences (Tukey’s HSD test, *P* ≤ 0.01). (*C*) The violin plots show transcriptional changes of 1,146 and 910 genes with low (≦0.4) and high (>0.4) IPC, respectively (see text), after 6, 24, and 48 h postinoculation (hpi) relative to vegetative mycelia. The horizontal line and closed circle in the boxplots represent the median and the average, respectively.

To examine the role of H3K27 modification in the regulation of HGT candidates, we performed RNA-seq analysis using a deletion mutant of *MoKmt6* encoding lysine methyltransferase responsible for H3K27me3 in *P. oryzae* (Pham et al. 2015). In vegetative mycelia, 960 and 918 genes were, respectively, up- and down-regulated (*P* < 0.01, fold change >2) in Δmokmt6. Interestingly, when *P. oryzae* genes were categorized into six groups based on IPC values, a gene group with lower IPC values tended to show a higher average of up-regulation levels in Δmokmt6 (fig. 7*A*), suggesting that HGT-derived genes are preferentially repressed by H3K27me3 in vegetative mycelia. In support of this hypothesis, the average levels of H3K27me3 in the 6 groups increased as their IPC levels decreased (fig. 7*B*). Although not as markedly as H3K27me3, average levels of H3K9me3 were also slightly elevated as IPC levels decreased (fig. 7*B*).

To ask if the HGT candidates repressed by H3K27me3 can be activated in response to some environmental signals, their expression levels during infection were examined using our RNA-seq data together with data in public databases (DRR496981 and DRR496982). We focused on 1,146 and 910 genes that had a low (≦0.4) and high (>0.4) IPC, respectively, and showed a low expression level (<5 TPM [transcripts per million]) in vegetative mycelia. While approximately half of the low IPC genes remained at low expression levels (<5 TPM), 10–20% showed more than 10 fold up-regulation and 2–3% showed more than 100 fold up-regulation at any time point during infection (fig. 7*C*). The rates of up-regulation were comparable between the low and high IPC genes. These data suggest that at least a fraction of HGT-derived genes are not only repressed by H3K27me3 but now contribute to the establishment of the fungal niche in response to environmental signals as efficiently as the original own genes.

## Discussion

### Means for Generating Genetic Variation in *Pyricularia* Fungi

Asexual reproduction appears to be a widespread mode of reproduction in phytopathogenic fungi. The sexual stage of *P. oryzae* was unknown until the 1970s (Ueyama and Tsuda 1975; Kato, et al. 1976; Yaegashi and Nishihara 1976). Some *P. oryzae* strains are now known to be fertile under experimental conditions, but the sexual stage of *P. oryzae* has never been observed in the field. Thus, it is believed that the infection cycle of *P. oryzae* is mostly asexual. How *P. oryzae* produces genetic variation not only for adaptation to host plants but also under various environmental conditions is an important question. This study showed that in *P. oryzae,* at least two different means were used to achieve this.

First, SNP analysis indicated that the genome of Br48 was generated by chromosomal crossover between *Brachiaria* and an ancient MoT or MoL strains (fig. 1), providing strong support for the hypothesis that *P. oryzae* undergoes genetic recombination under natural conditions by a sexual cross or parasexual recombination. Gladieux et al. (2018) performed a large-scale analysis of the population structure of *P. oryzae* using whole-genome sequence information for 76 *P. oryzae* isolates sampled from 12 grass and cereal genera and established several discrete lineages in the *P. oryzae* population, each infecting a limited number of host species. Interestingly, their analysis indicated the existence of gene flow and admixture among multiple *P. oryzae* lineages. They proposed that “universal suscepts” such as barley, Italian ryegrass, tall fescue, and weeping lovegrass, which are host plants susceptible to a variety of *P. oryzae* isolates from different lineages (Kato, et al. 2000), serve as a common place for *P. oryzae* isolates to encounter and mate with each other. It is important to note that the genomic region derived from the *Brachiaria* strain contains the virulent B-type *PWT3* allele. *PWT3* is one of the two virulent loci responsible for the recent emergence of MoT, likely from the MoL (Inoue, et al. 2017). Thus, chromosomal recombination may be an important contributing factor to the emergence of a new pathogen in *P. oryzae*.

Second, the Br48 genome contains a large number of putative HGT-derived genes. Previously it was suggested that the avirulence gene, *AVR-Pita2* was horizontally transferred between two cross-sterile *Pyricularia* species, *P. grisea*, and *P. oryzae* (Chuma, et al. 2011). This study suggests that gene flow between *P. grisea* and *P. oryzae* occurs at a megabase scale. Approximately 0.22 Mb and 0.24 Mb DNA segments on chromosome 3 showed sequence similarity and synteny to chromosome segments of *P. pennisetigena* and *P. grisea* (fig. 2*B*), whereas those segments were absent or only partially conserved in the MoO and MoE strains of *P. oryzae*. Thus, megabase-scale DNA fragments were likely horizontally transferred among *Pyricularia* fungi. In addition, IPC and phylogenetic analyses in this study indicated that some HGT candidates in Br48 were derived from distantly related Ascomycete fungi. Such asexual exchange of genetic materials should have contributed to the genetic variation in the *Pyricularia* population.

### Mini-Chromosomes and Interchromosomal Translocation

Based on the comparison of genome structure among the *P. oryzae* isolates (supplementary fig. 1S, Supplementary Material online), it was assumed that megabase-scale (0.36 and 0.21 Mb) DNA fragments were inserted into chromosomes 6 and 7 of the Br48 genome. These insertions were not observed in the genome of another MoT strain, B71, and thus, should have occurred recently on the evolutionary time scale. It should be noted that such megabase-scale insertions appeared to have occurred in all *P. oryzae* isolates examined at different locations in the genome (supplementary fig. 1S, Supplementary Material online). Interestingly, the 0.36 Mb insertion shared a partial sequence similarity, albeit highly rearranged, to the mini-chromosome in the B71 strain, suggesting that the insertion originated from a mini-chromosome by interchromosomal translocations between core- and mini-chromosomes, as proposed in *P. oryzae* by Langner et al. (2021). Our data indicate that a mini-chromosome could be integrated into a core chromosome (CC) after mini-chromosome generation with complex rearrangements. In this context, it should be noted that Br48 does not possess mini-chromosomes, which might be because the former mini-chromosome(s) had been integrated into CCs in Br48.

The term mini-chromosome implies that its physical size is smaller than that of normal chromosomes. Mini-chromosomes are also called “conditionally dispensable,” “supernumerary,” or “accessory” chromosomes (AC) since these chromosomes are often not essential. However, ACs can be beneficial under certain conditions by carrying genes for producing a phytotoxin, an effector protein and detoxifying host defense compounds (Han, et al. 2001; Hatta, et al. 2002; Ma, et al. 2010; Mehrabi, et al. 2011).

Fungal ACs appear to share certain specific features, such as enrichment of repetitive sequences, low gene density, different codon usage compared to CCs, silent histone modifications H3K9me3 and H3K27me3, and higher mutation rates than CCs (Coleman et al. 2009; Ma et al. 2010; Goodwin et al. 2011; Connolly et al. 2013; Schotanus et al. 2015; Habig et al. 2021). In *Fusarium* and *Zymoseptoria*, certain large segments on CCs exhibit AC signatures, typically high H3K27me3 accumulation (Connolly et al. 2013; Schotanus et al. 2015). Erlendson et al. (2017) proposed that such large AC-like segments occur either by the spread of subtelomeric H3K27me3-enriched regions toward the centromere or by interchromosomal translocations between accessory and core chromosomes.

Our comparative genomic analysis indicated that most of the AC-like large segments in the Br48 genome appeared to have occurred by insertional events of a large DNA segment or translocations with a complex rearrangement, supporting the latter model. In fact, the 0.36 Mb “insert” on chromosome 6 shared a partial sequence similarity with a mini-chromosome as mentioned above. In this model, large segments in CCs are marked with H3K27me3 because they are derived from ACs. Once integrated into CCs, ACs become AC domains on CCs, and are marked with H3K27me3.

### Epigenetic Modifications of Horizontally Transferred DNA

Based on IPC analysis, many of the HGT candidates in the Br48 genome were detected in specific regions of the genome, typically in VDs including large “insertions”. Thus, as discussed above, these genes may have been gained via ACs by interchromosomal translocation. In fact, ACs are mostly transmitted to offspring in a non-Mendelian manner and are reported to be horizontally transferred between distantly related fungi (Mehrabi, et al. 2011). Thus, it is tempting to hypothesize that ACs serve as a molecular vehicle to transfer DNA between fungal species, similar to plasmids in prokaryotes, and also as a melting pot in which foreign DNA and chromosomal DNA may be mixed with each other.

Interestingly, the genomic locations of HGT candidates are often associated with H3K9me3 or H3K27me3 (fig. 6*A*; supplementary figs. S6–S7, Supplementary Material online), suggesting that HGT-derived genes are generally targeted for silent epigenetic modifications. In mammals, H3K27me3 was shown to be associated with double strand DNA breaks (DSB) (O'Hagan et al. 2008; Chou et al. 2010). It appears that DSB during the integration of an HGT-derived gene into either CCs or ACs may induce H3K27me3 also in fungi.

On chromosomes 2, 5, and 6, we detected H3K27me3 enrichment in the Br48 genomic regions that did not correspond to either a subtelomeric region or any location of a large rearrangement between the Br48 and 70-15 strains. This may be explained by assuming that these domains originated from an ancient AC that contained HGT-derived genes and was integrated into CCs before the diversification between MoT and MoO. Phylogenetic analysis of resident genes supported this model (fig. 6*B*). Alternatively, these domains are native facultative heterochromatin marked with H3K27me3, which accelerates the rate of spontaneous mutations (Habig et al. 2021), leading to low sequence conservation that may affect IPC. In our analysis, H3K27me3 was found to be associated with VDs. While large VDs mostly consist of megabase-scale insertions and translocations, small VDs often differ only in sequence with frequent base substitutions, small insertions, and small deletions. Thus, H3K27me3 may be associated with high rates of spontaneous mutations in *P. oryzae*. In this context, IPC is not considerably affected by high mutation rates in a single fungal species but can be biased if the same happens to the corresponding orthologous genes in the majority of the fungal species examined. In fact, some of decreased IPC values may be attributed to high mutation rates in H3K27me3-rich regions. However, it is highly unlikely that such mutations made *P. oryzae* sequences similar to the ones in distantly related organisms such as basidiomycetes or bacteria.

Overall, our comparative genomics and IPC analyses support the hypothesis that the Br48 genome contains many HGT-derived genes associated with H3K27me3, a silent histone mark for an “accessory” gene or region of the genome. An HGT-derived gene should be accessory at the beginning because the recipient organism had been living without it before the acquisition. Thus, gene suppression by H3K27me3 on an AC or AC domain might occur as an initial defense mechanism against foreign genes (Van Vu et al. 2021) whose expression could be harmful to the recipient, and later some of the genes become domesticated to be expressed under host control in response to environmental signals, as suggested in this study.

## Materials and Methods

### High-Throughput Sequencing of Fungal DNA and RNA

The wheat blast fungus Br48 strain was collected in Brazil in 1990 (Urashima et al. 1993) and kept on barley seeds media at 4° for long-term storage (Nakayashiki, et al. 1999). For the working culture, a barley grain from the stock culture was placed on a PDA (potato dextrose agar) slant media and cultured at 25°. For DNA and RNA extraction, fungal plugs were transferred to flasks containing complete medium (CM) (5% sucrose, 3% casamino acids, and 3% yeast extract) and incubated in a shaker at 120 rpm at 25° for 4 days. A CTAB (cetyltrimethylammonium bromide) DNA extraction method was used to isolate genomic DNA for sequencing (Talbot et al. 1993). Fungal RNA was extracted using Sepasol RNA I SuperG (Nacalai Tesque) as described previously (Nguyen et al. 2018) and further cleaned using the NucleoSpin RNA Clean-up Kit (Macherey–Nagel, Düren, Germany). Ribo-Zero Gold rRNA Removal Kit (Human/Mouse/Rat) (Illumina, San Diego, USA) was used for rRNA depletion.

For MiSeq sequencing, genomic DNA libraries were constructed using NEBNext Ultra DNA Library Prep Kit for Illumina. Paired-end sequencing was performed using the 600-cycle (MiSeq Reagent Kit v3) sequencing kit format. For HiSeq (HiSeq 2500 and HiSeq X) and Pacbio (PacBio RS II) sequencing, libraries were prepared using kits provided by the manufacturer of the sequencing platform at Macrogen Japan Co. Ltd (Kyoto, Japan) and Takara-bio Co. Ltd (Otsu, Japan).

### Genome Assembly and Annotation

The initial draft assembly was built from 3.5 GB PacBio long reads using the HGAP at DDBJ (www.ddbj.nig.ac.jp). Assembly polishing was performed using the Illumina reads. In addition, the de novo assembly of short reads from HiSeq and MiSeq platforms together with publicly available short read data of Br48 were performed with CLC Genomics Workbench ver. 11.0.1. The HGAP unitigs were joined using the short read derived contigs. When a gap between HGAP unitigs was estimated to be less than 20 kb based on reference genomes in public databases, long PCR was performed to confirm the contiguity and distance between the unitigs using KOD-FX (TOYOBO, Osaka, Japan) and GoTaq Long PCR Master Mix (Promega, Madison, USA). The gap was manually filled using PacBio long reads based on the sequence similarity to the reference sequences.

Repeat sequences in the Br48 genome were first detected by RepeatMasker v4.0.3. Among them, TEs were defined manually in reference to typical signatures of TEs such as transposase, long terminal repeats, and reverse transcriptase together with their distribution patterns in the genome. We also refer to a list of TEs in the wheat isolate of *P. oryzae*, B71 (Peng, et al. 2019).

Gene prediction and functional annotation were performed with the Funannotate v1.8.10 (https://github.com/nextgenusfs/funannotate) pipeline. Our RNA-seq data together with transcripts and proteins from related *Pyricularia* genomes in NCBI database (https://www.ncbi.nlm.nih.gov/protein/? term = Pyricularia_oryzae) were used to support gene prediction.

### Chromatin Immunoprecipitation-Seq Analysis

ChIP assay was performed using the ChIP-IT Express kit (Active Motif, Carlsbad, USA) following the manufacturer's protocol as described previously (Pham, et al. 2015). Fungal mycelia were grown in CM liquid for 4 days at 26° on an orbital shaker (120 rpm). A portion of mycelia (50 mg) was harvested and incubated at room temperature for 15 min in 10 ml of phosphate-buffered saline (PBS) containing formaldehyde at a concentration of 1%. Chromatin was sheared by sonication using a Bioruptor apparatus (Cosmo Bio Co., Ltd., Japan) for three cycles of 1 min on at high intensity (200 W) and 30 s off, followed by five cycles of 1 min on at medium intensity (160 W) and 30 s off. The size of the sheared chromatin was around 100 to 500 bp as determined by agarose gel electrophoresis. Antibodies against demethylated H3 Lys 4 (Active Motif, #39141), trimethylated H3 Lys9 (Active Motif, #39161), and trimethylated H3 Lys27 (Active Motif, #39156) were obtained from Active Motif. ChIPed DNA was recovered by phenol-chloroform extraction and ethanol precipitation. Libraries for high-throughput sequencing were constructed using NEBNext Ultra II DNA Library Prep Kit for Illumina and sequenced using HiSeq X at Macrogen Japan Co. Ltd (Kyoto, Japan).

### Mapping, SNP, and Comparative Genomics Analyses

RNA-seq and ChIP-seq reads were mapped to the Br48 genome using CLC Genomics Workbench ver. 11.0.1 with parameter settings, length fraction = 0.9 and similarity fraction = 0.9 unless otherwise mentioned. For SNP analysis, Illumina short reads of the *Brachiaria* strain (Br35) and the wheat strains (Br116.5 and Br118.2) were obtained from public databases and mapped using parameter settings, fraction = 0.8 and similarity fraction = 0.7. SNP calls were made using the following settings; ploidy = 1, minimum coverage = 10, minimum count = 8, minimum frequency = 90%, neighborhood radius = 5, minimum central quality = 20, and minimum neighborhood quality = 20. The data of Br116.5 was used for chromosomes 2 and 5, and those of Br118.2 was used for the other chromosomes in figure 1. To visualize comparisons between multiple *P. oryzae* genomes, the Easyfig software (Sullivan et al. 2011) was used.

### Index of Phylogenetic Concordance

To calculate IPC, twenty Ascomycete fungal species were selected, which included twelve Sordariomycetes fungi, *Pyricularia grisea*, *Magnaporthiopsis poae* (ATCC 64411), *Gaeumannomyces tritici* (R3-111a-1), *Podospora comate*, *Colletotrichum orbiculare* (MAFF 240422), *Cryphonectria parasitica* (EP155), *Valsa mali* var. *pyri*, *Xylaria grammica*, *Fusarium oxysporum* f. sp. *cubense*, *Hirsutella minnesotensis* (3608), *Metarhizium brunneum* (ARSEF 3297), and *Trichoderma reesei* (QM6a), three Leotiomycetes fungi, *Sclerotinia sclerotiorum* (1980 UF-70), *Botrytis cinerea* (BcDW1), and *Pseudogymnoascus* sp. (WSF 3629), two Eurotiomycetes fungi, *Penicillium nordicum*, and *Aspergillus niger* (ATCC 1015), and three Dothideomycetes fungi, *Bipolaris maydis* (ATCC 48331), *Pyrenophora tritici-repentis* (Pt-1C-BFP), and *Alternaria atra*. Their phylogenetical species relationship was first examined using seven conserved genes, which included DNA-directed RNA polymerase I subunit RPA2, elongation factor G (EF-G), elongation factor 1 ɑ (EF1- ɑ), tubulin beta chain (Tub2), heat shock protein 70 (HSP70), RNA polymerase II (RNAPII), and actin. The ranking of species similarity to *P. oryzae* was made according to a sum of BLASTP scores of the top hit proteins in a fungal species against the seven conserved proteins in *P. oryzae*. Consequently, the species ranking was determined in order of their appearance in this section.

The ranking of gene similarity to a gene in *P. oryzae* was made according to BLASTP scores of the top-hit protein in the twenty fungal species. When no orthologous proteins were detected in fungal species by BLAST search with the cutoff values of E-value <0.01 and query cover >40%, the average of the lower ranks was given to all of them. For example, if five fungal species do not possess a detectable ortholog, they are all ranked as 18th. When an ortholog was not detectable in more than 10 fungal species, the gene was eliminated from the analysis. The IPC of a gene was given as a correlation coefficient between the rankings of species similarity and gene similarity.

### Phylogenetic Analysis

Homologs of a Br48 gene were searched by the BLASTP or BLASTX program with default parameters. Homologs from different Order, Class, or higher taxonomic rank were preferentially included in the analysis. To reduce false positives due to contamination, the following two conditions were imposed when homologs were included in the analysis: 1) at least two examples are detected in organisms of the same taxonomic category, and 2) homologs that are found repeatedly in the same species outside of Ascomycetes, are excluded from the analysis. Amino acid sequences of selected homologs were aligned using the ClustalW program under default parameters using MEGA X (Kumar et al. 2018). The phylogenetic reconstructions were performed using the neighbor-joining method implemented in MEGA X with a statistical confidence of 1,000 replicates. The tree topologies were also confirmed with the maximum-likelihood method.

## Supplementary Material

msad186_Supplementary_Data

## Data Availability

The telomere-to-telomere genome sequence of the Br48 strain was deposited at DDBJ/EMBL/GenBank under the accession numbers AP027063- AP027069. RNA-seq and ChIP-seq read of the Br48 strain have been deposited on DDBJ Sequence Read Archive under the accession numbers PRJDB2912, PRJDB3851, and DRA016397.
