# Supplementary material for: Horizontally Transferred DNA in the Genome of the Fungus Pyricularia oryzae is Associated With Repressive Histone Modifications
Source: Mol Biol Evol. 2023 Aug 18;40(9):msad186. doi: 10.1093/molbev/msad186 (PMC10473863; doi:10.1093/molbev/msad186)
Supplement: msad186_Supplementary_Data [file msad186_supplementary_data.zip › SI_Fig.pdf]

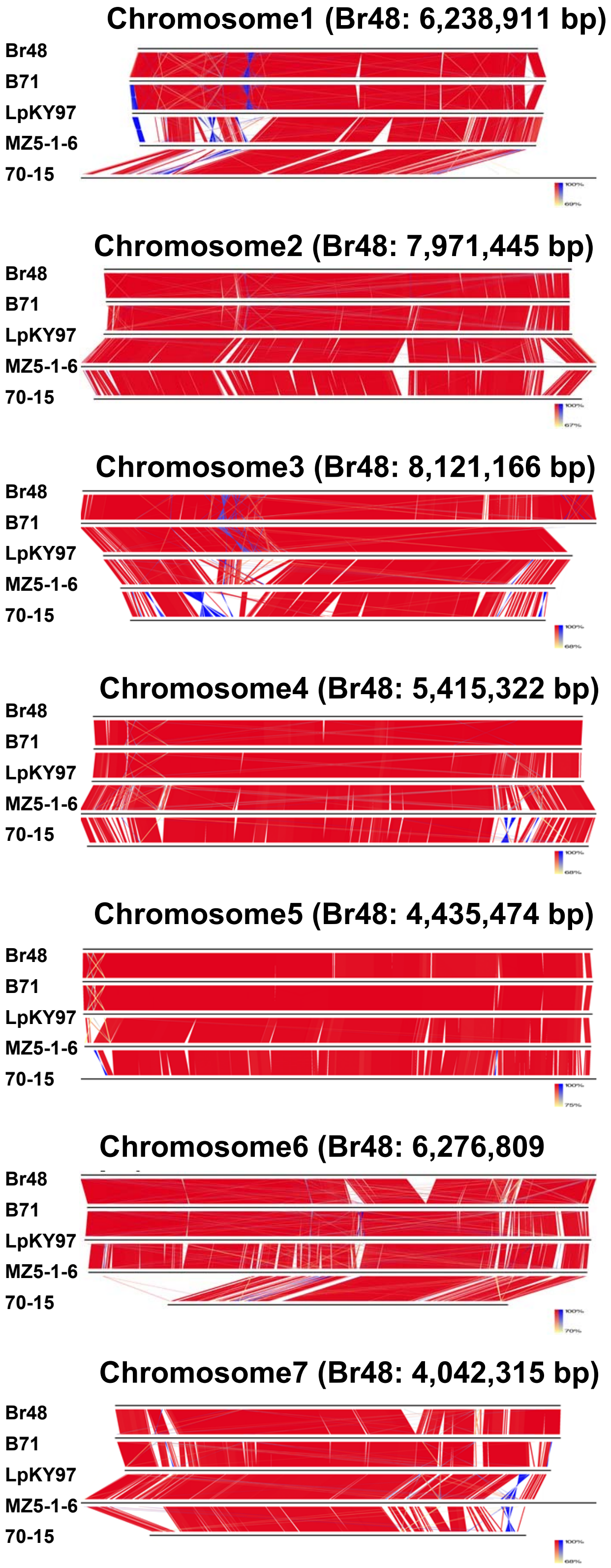

Fig. S1. Comparison of chromosome structure among five *Pyricularia oryzae* strains, Br48 (MoT), B71 (MoT), LpLY97 (MoL), MZ5-1-6 (MoE), and 70-15 (MoO). BLAST comparison was visualized by the Easyfig software (Sullivan et al. 2011).

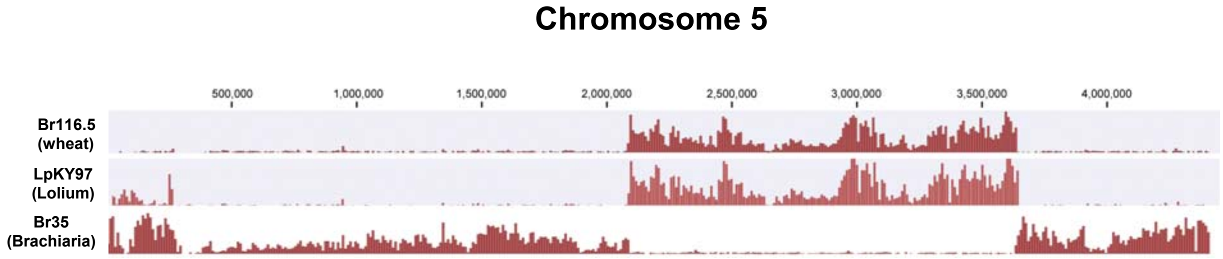

Fig. S2. SNP distribution on chromosome 5 of Br48. Panels show SNPs identified by a comparison with wheat (Br116.5)-, *Lolium* (LpKY97)- and *Brachiaria* (Br35)-infecting strains, respectively.

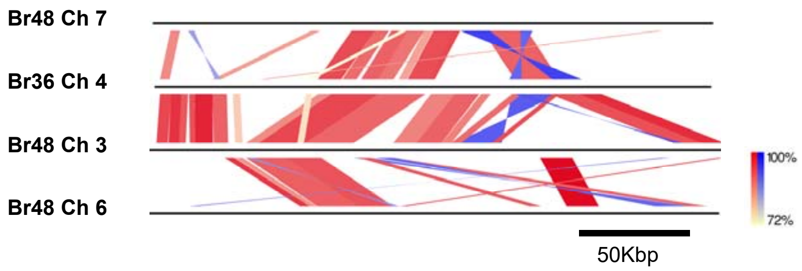

Fig. S3. Syntenic regions found in mega-scale "insertions" in the Br48 genome. An approximately 40 kb sequence syntenic to a *P. pennisetigena* genome segment (Ch4_#562,900-#764,500) was present in three megabase-scale "insertions" on chromosome 3 (#1,509,300-#1,720,400), 6 (#4,029,500-#4,236,800), and 7 (#2,599,300-#2,802,300) in the Br48 genome.

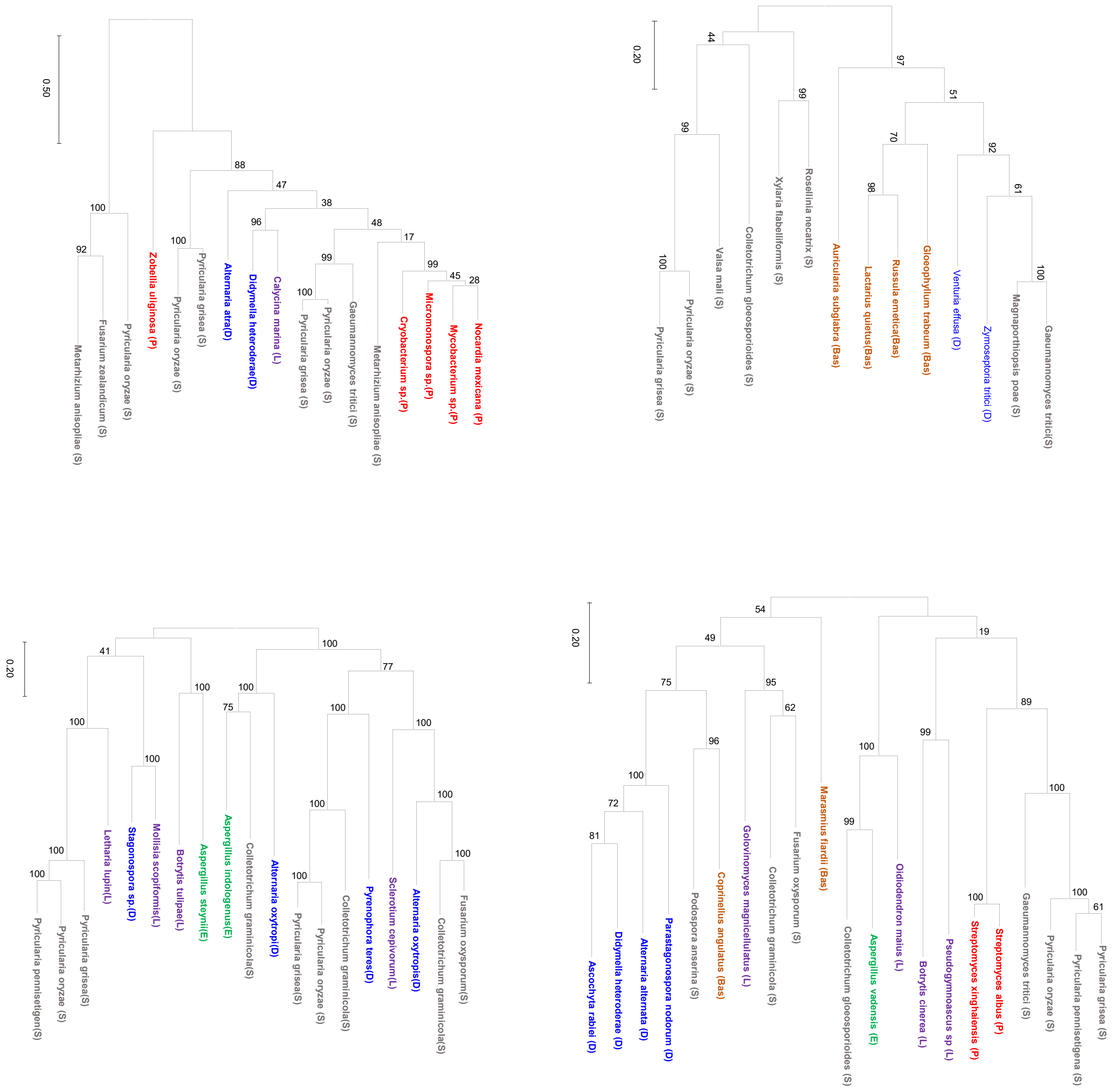

Fig. S4. Maximum likelihood trees of (*A*) FAD/FMN-binding dehydrogenase and (*B*) putative small secreted protein shown in Fig. 3 together with (*C*) PKS-NRPS hybrid protein and (*D*) galactose oxidase protein shown in Fig.6B. Accession numbers of protein sequences are given in Fig. 3 and Fig.6B. Bas, Basidiomycetes (shown in Brown); E, Eurotiomycetes (green); D, Dothideomycetes (Blue); L, Leothiomycetes (purple); P, Prokaryotes (red); S, Sordariomycetes (gray); Sc, Schizosaccharomyces (pink).

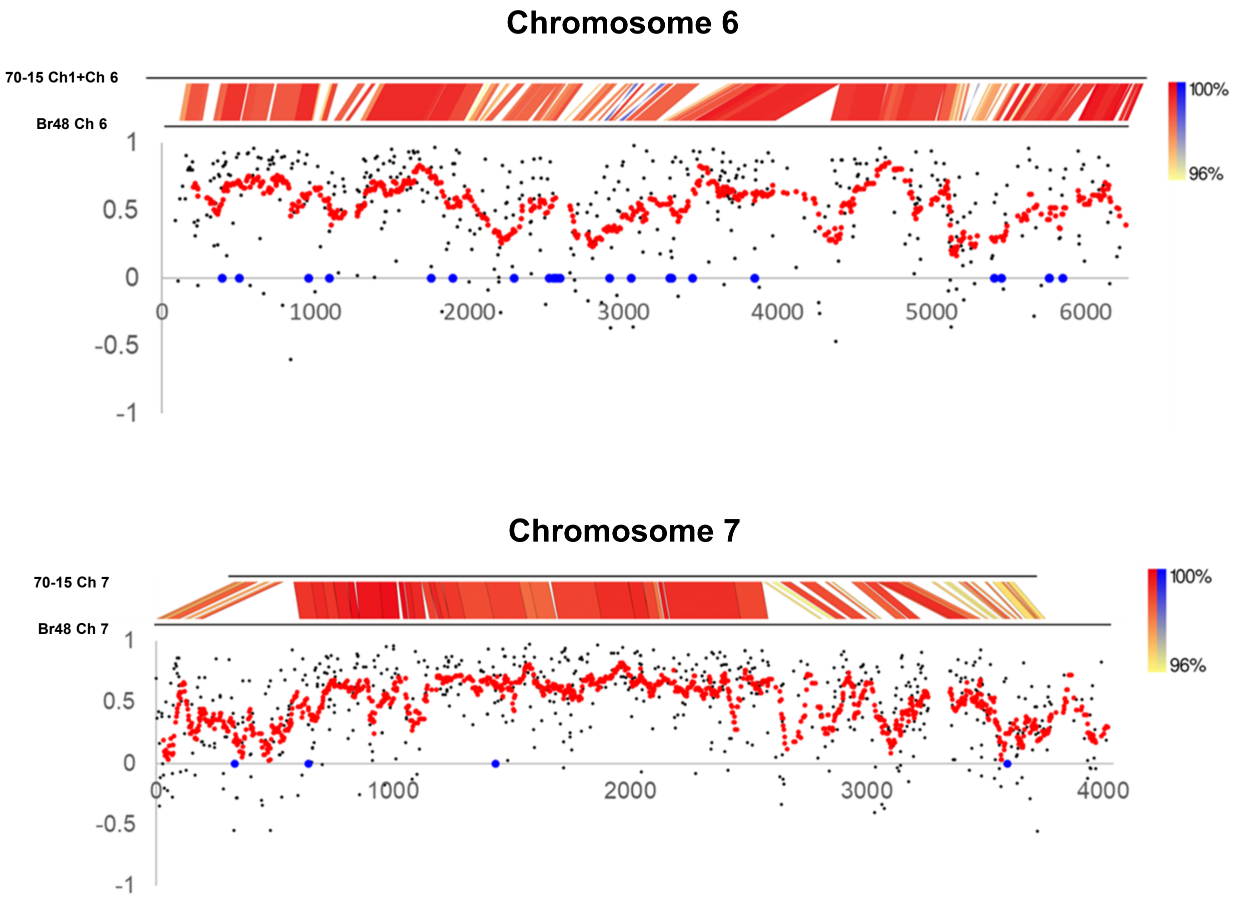

Fig. S5. Index of phylogenetical concordance (IPC) values on chromosomes 6 and 7. Since a part of chromosome 6 in the 70-15 strain was translocated to chromosome 1, the corresponding region in chromosome 1 was added to chromosome 6 of the strain for a comparison of genome structure. Black dots represent IPC values of individual genes. Red dots indicate moving averages of 15 consecutive IPC values. The genomic positions of *P. oryzae* homologs to possible HGT genes identified by Qiu et al. (2016) are shown as blue dots.

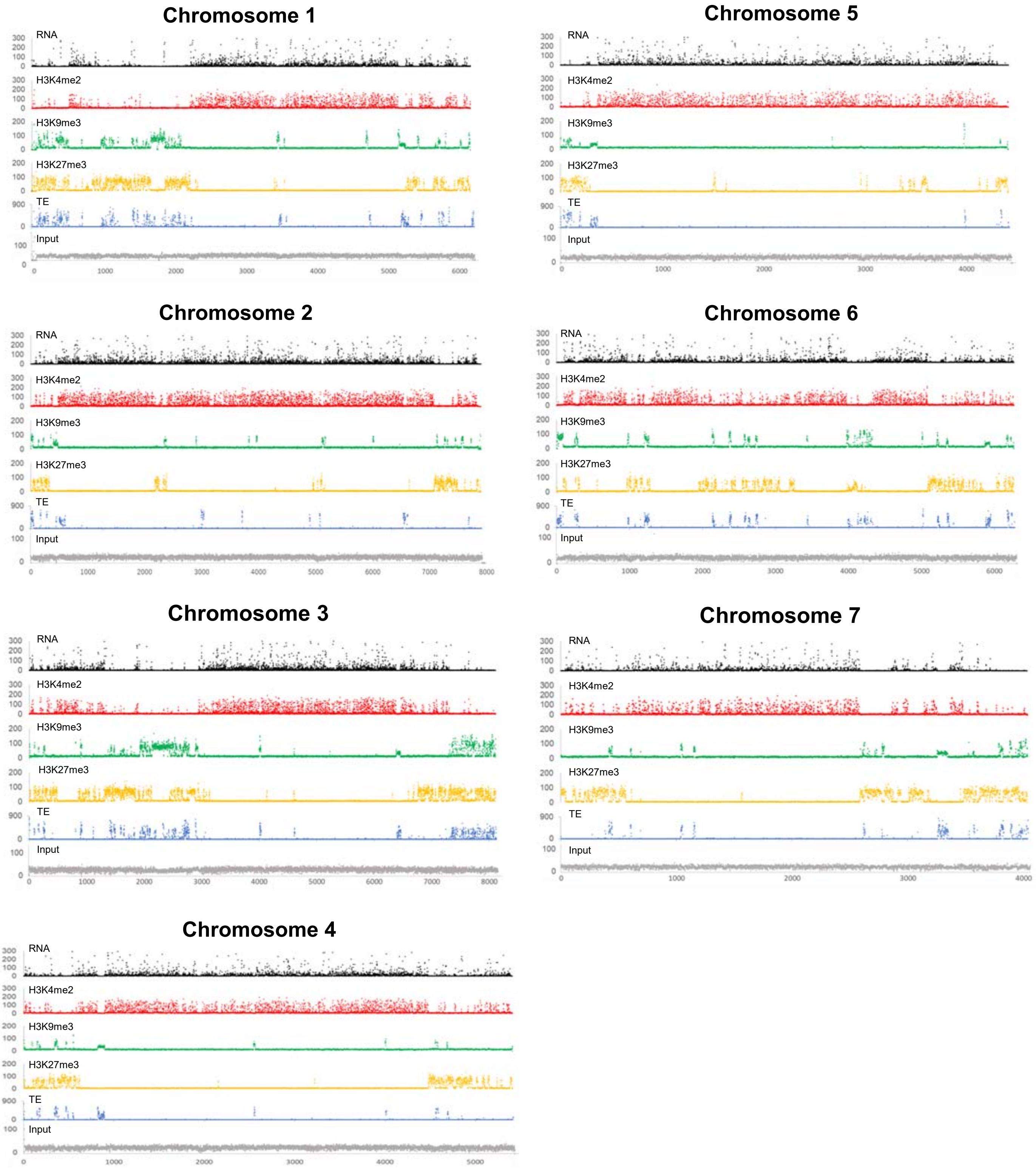

Fig. S6. Read mapping data of RNA-seq and ChIP-seq analysis on Br48 chromosomes. RNA used in the analysis was extracted from vegetative mycelia. Reads mapped to TE sequences (Table S2) were collected and then, mapped to the Br48 genome. Sonicated DNA was used as an input control, Each dot indicates an RPM value of a 1kb segment in the Br48 genome (see the text).

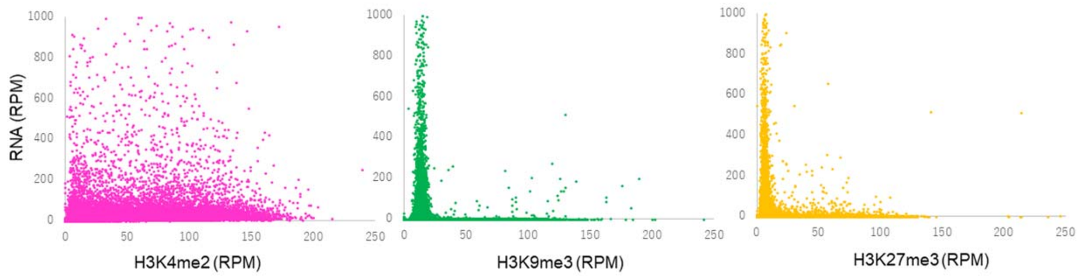

Fig. S7. Relationship between RNA expression and histone modifications RPM values of RNA-seq (Y-axis) and ChIP-seq (X-axis) analyses in a 1 kb segment were plotted as a dot. Antibodies against H3K4me2, H3K9me3 and H3K27me3 were used in ChIP-seq analyses.

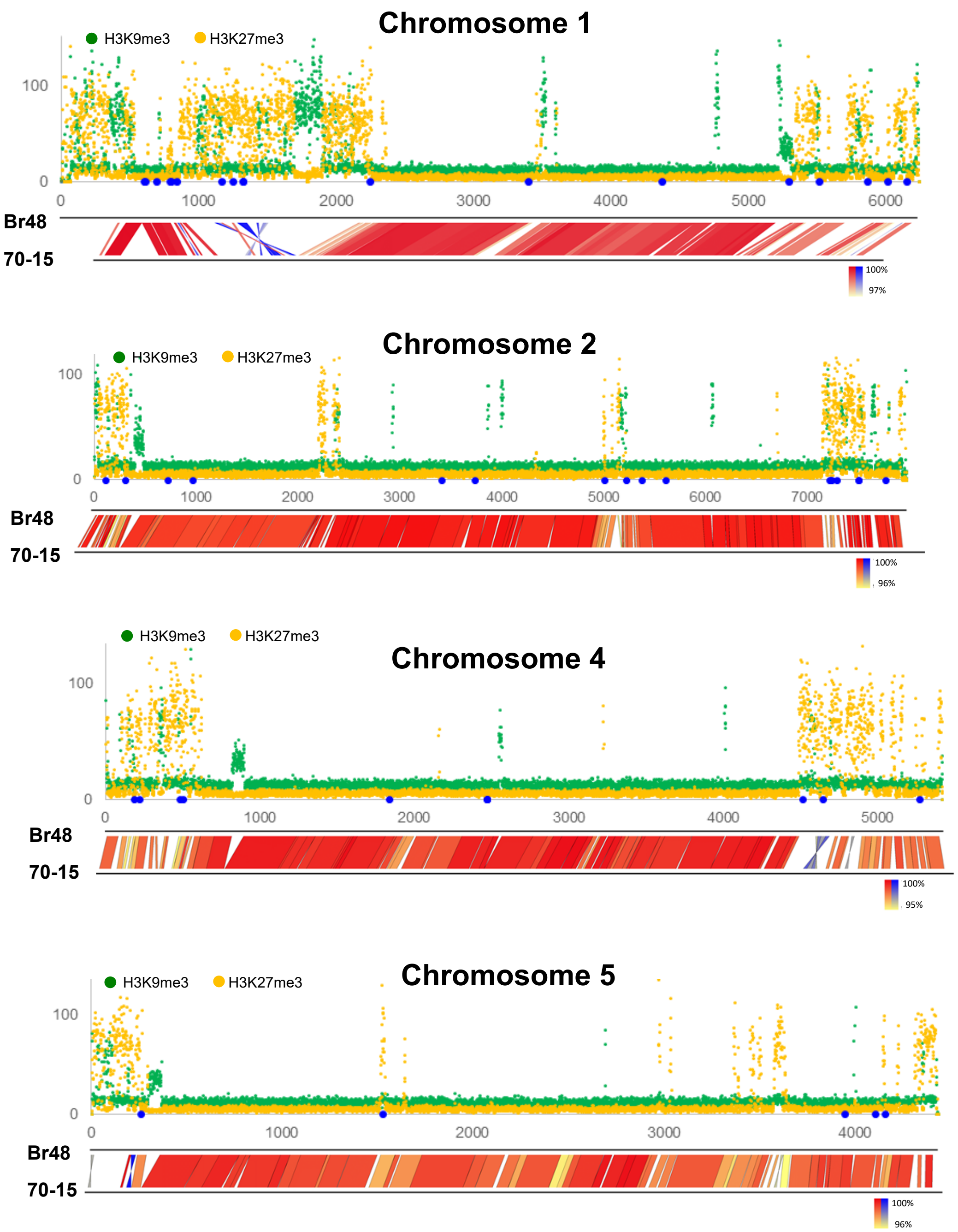

Fig. S8. Read mapping of H3K9me3 and H3K27me3 on chromosomes 1, 2, 4 and 5 of Br48. A comparison of chromosome structure between the Br48 and 70-15 strains are shown below the mapping data. The genomic positions of *P. oryzae* homologs to possible HGT genes identified by Qiu et al. (2016) are shown as blue dots

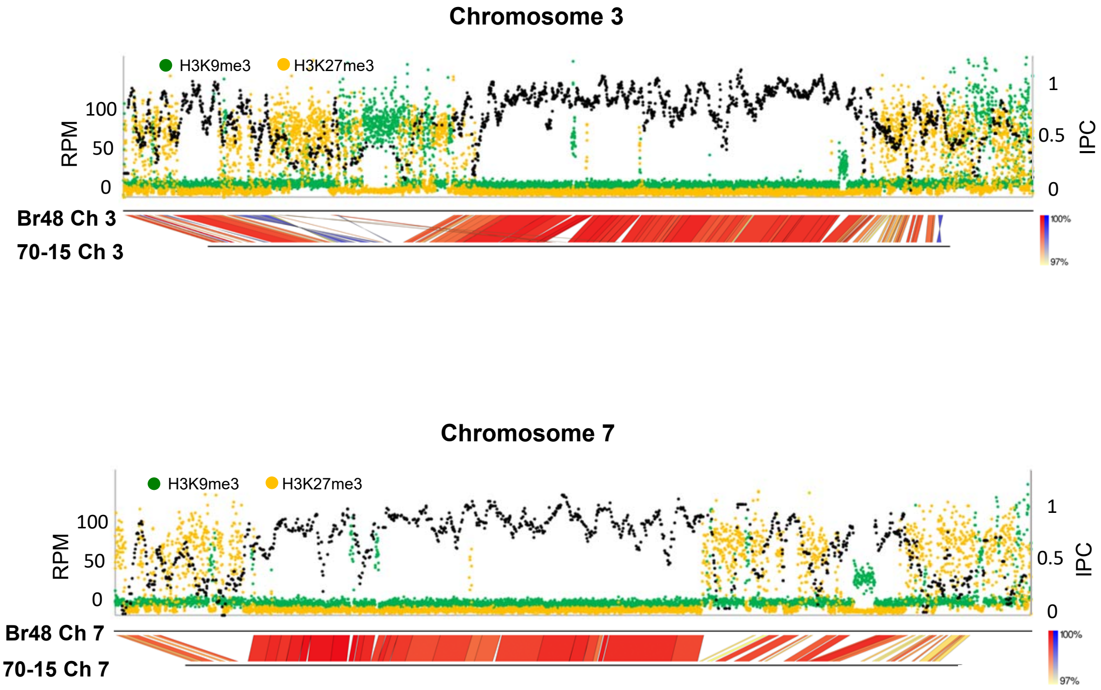

Fig. S9. Read mapping of H3K9me3 and H3K27me3 on chromosomes 3 and 7 of Br48. The black dots show moving averages of 15 consecutive IPC values on the chromosomes. A comparison of chromosome structure between the Br48 and 70-15 strains are shown below the mapping data.
